# Modeling positional effects of regulatory sequences with spline transformations increases prediction accuracy of deep neural networks

**DOI:** 10.1093/bioinformatics/btx727

**Published:** 2017-11-16

**Authors:** Žiga Avsec, Mohammadamin Barekatain, Jun Cheng, Julien Gagneur

**Affiliations:** 1 Department of Informatics, Technical University of Munich, Garching, Germany; 2 Graduate School of Quantitative Biosciences (QBM), Gene Center, Ludwig-Maximilians-Universität München, Munich, Germany

## Abstract

**Motivation:**

Regulatory sequences are not solely defined by their nucleic acid sequence but also by their relative distances to genomic landmarks such as transcription start site, exon boundaries or polyadenylation site. Deep learning has become the approach of choice for modeling regulatory sequences because of its strength to learn complex sequence features. However, modeling relative distances to genomic landmarks in deep neural networks has not been addressed.

**Results:**

Here we developed spline transformation, a neural network module based on splines to flexibly and robustly model distances. Modeling distances to various genomic landmarks with spline transformations significantly increased state-of-the-art prediction accuracy of *in vivo* RNA-binding protein binding sites for 120 out of 123 proteins. We also developed a deep neural network for human splice branchpoint based on spline transformations that outperformed the current best, already distance-based, machine learning model. Compared to piecewise linear transformation, as obtained by composition of rectified linear units, spline transformation yields higher prediction accuracy as well as faster and more robust training. As spline transformation can be applied to further quantities beyond distances, such as methylation or conservation, we foresee it as a versatile component in the genomics deep learning toolbox.

**Availability and implementation:**

Spline transformation is implemented as a Keras layer in the CONCISE python package: https://github.com/gagneurlab/concise. Analysis code is available at https://github.com/gagneurlab/Manuscript_Avsec_Bioinformatics_2017.

**Supplementary information:**

Supplementary data are available at *Bioinformatics* online.

## 1 Introduction

In recent years, deep learning has proven to be powerful for modeling gene regulatory sequences. Improved predictive accuracies have been obtained for a wide variety of applications spanning the modeling of sequences affecting chromatin states (Kelley *et al.*, 2016; Zhou and Troyanskaya, 2015), transcription factor binding (Alipanahi *et al.*, 2015), DNA methylation (Angermueller *et al.*, 2017) and RNA splicing (Leung *et al.*, 2014; Xiong *et al.*, 2015), among others. Using multiple layers of non-linear transformations, deep learning models learn abstract representations of the raw data and thereby reduce the need for handcrafted features. Moreover, the deep learning community, which extends much beyond the field of genomics and includes major web companies, is actively developing excellent software frameworks that allow rapid model development, model exchange and scale to very large datasets (Abadi *et al.*, 2016; Bastien *et al.*, 2012; Collobert *et al.*, 2002; Jia *et al.*, 2014). Altogether, it is advantageous to leverage these strengths and further develop deep learning modules specific for regulatory genomics.

The distance to defined locations in genes such as transcription start site (TSS), start codon, stop codon, exon junctions or polyadenylation [poly(A)] site, which we refer to collectively as genomic landmarks, plays an important role in regulatory mechanisms. Genomic landmarks are often bound by regulatory factors. For instance, RNA 5ʹ ends are bound by capping factors, exon junctions by the exon junction complex and the poly(A)-tail by the poly(A)-binding proteins. These factors provide spatial clues for other factors to be recruited and to interact. Furthermore, distances to genomic landmarks can be important for structural reasons. The relatively well-defined distance between the TATA-box and the TSS is due to structural constraints in the RNA polymerase complex (Sainsbury *et al.*, 2015). Also, the splice branchpoints are typically localized within 18–44 nt of the acceptor site due to specific constraints of the spliceosome (Mercer *et al.*, 2015; Wahl *et al.*, 2009). Therefore, splice branchpoints are not only defined by their sequence but also by their distances to the acceptor site. This information can be used to improve prediction of branchpoint location from sequence (Bitton *et al.*, 2014; Corvelo *et al.*, 2010; Signal *et al.*, 2016).

Despite their important role in gene regulation and their successful usage in computational models, distances to genomic landmarks have not been included in deep learning models. Typical sequence-based deep learning models take into account the effects of relative position *within* each sequence (internal position), either by using strided pooling after convolutional layers followed by fully connected layers or by using weighted sum pooling (Shrikumar *et al.*, 2017). However, modeling effects of internal positions does not cover modeling of positions to genomic landmarks. These are defined externally to the sequence and can lie at very long distances, as in the case of enhancer to promoter distances. Additionally, genomic landmarks might be difficult to discover *de novo* by the model. While categorical genomic region annotation such as promoter, UTR, intron or exon capture relevant spatial information and help improving prediction performances (Pan and Shen, 2017; Stražar *et al.*, 2016), they are still not capturing distances to genomic landmarks quantitatively.

Here we demonstrate the importance of using relative distances to genomic landmarks as features in sequence-based deep learning models. Technically, we achieve this by introducing spline transformation (ST), a neural network module to efficiently integrate scalar features such as distances into neural networks. Spline transformation is based on smooth penalized splines (P-splines; Eilers and Marx, 1996) and can be applied both in the context of fully connected layers as well as convolutional layers. We show that deep neural networks (DNNs) modeling effects of distances to genomic landmarks outperform state-of-the art models on two important tasks. First, we obtain consistent improvements for predicting UV crosslinking and immunoprecipitation (CLIP) peaks across two datasets: a large enhanced CLIP (eCLIP) ENCODE dataset containing 112 RNA-binding proteins (RBPs) (Van Nostrand *et al.*, 2016) and a well-studied CLIP benchmark dataset (Pan and Shen, 2017; Stražar *et al.*, 2016) containing 19 RBPs from 31 experiments. Second, we obtain the best model for predicting splice site branchpoint (Mercer *et al.*, 2015). Furthermore, we show that across our applications, spline transformation leads to better predictive performance, trains faster and is more robust to initialization than piecewise linear transformations (PLTs), an alternative class of functions based on the popular rectified linear units (ReLUs).

## 2 Materials and methods

### 2.1 Spline transformation

#### 2.1.1 Definition

**Fig. 1.**
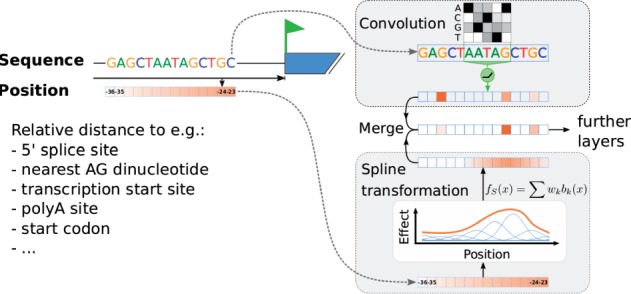
Simplified sketch of a model architecture using spline transformation. In addition to DNA sequence, relative distances to various genomic landmarks are used as features. Spline transformation [Equation (1)] learns a smooth transformation of the raw distances. Transformed distances are then merged with sequence-based activations of convolutional layers

We considered input data that not only consist of one-hot-encoded sequence vectors but also of scalar vectors. One typical and simple case is where each input consists of a nucleic acid sequence and a scalar vector of the same length containing the distance of every nucleotide to a genomic landmark of interest (Fig. 1). Another case is to have a single value per input sequence, for instance encoding the distance of the sequence midpoint to a genomic landmark. A single value per sequence may be appropriate when positional effects vary over much longer scales than the length of the sequence.

The positional effects are modeled with a smooth transformation function. We used P-splines or penalized splines (Eilers and Marx, 1996). Spline transformation *f_S_* is defined as

(1) $$f_{S}(x)=\sum_{k=1}^{B}w_{k}b_{k}(x;p),$$

where *b_k_* is the *k*th B-spline basis function of degree p∈ℕ (De Boor, 1978) (Fig. 1) and *x* is a multi-dimensional array of positions. In all the applications presented here we used cubic splines, i.e. *p* = 3. Spline bases are non-negative functions with finite support. Knots of the spline basis functions $\left\{b_{1},\ldots ,b_{D}\right\}$ are placed equidistantly on the range of input values *x*, such that the following relation holds:

(2) $$\;\sum_{k=1}^{B}b_{k}(x;p)=1\;\forall x,p.$$

The only trainable parameters in spline transformation are $w_{1},\ldots ,w_{B}$.

To favor smooth functions, a smoothness regularization is added to the global loss function:

(3) $$\text{regularization}(w)=\lambda w^{T}Sw,$$

where **S** is a symmetric positive matrix effectively encoding the squared second-order differences of the coefficients **w**, which approximate the square of second-order derivatives (Eilers and Marx, 1996). The advantage of this approach is that one can have finely spaced bases and use the regularization parameter *λ* to set the amount of smoothing.

#### 2.1.2 Integration into neural networks

Spline transformation is applicable at the network input values. In that case, the approximate range of values—a necessary requirement for the knot placement—is known. How and where the output of spline transformation is merged into the network is application specific. In the case of single values per sequence, the transformed values are added to the flattened output of the last convolutional layer, right before the fully connected layers (Supplementary Fig. S1a). In the case of scalar vectors along the sequence, their spline-transformed values are typically merged with the output of the first sequence-based convolutional layer (Supplementary Fig. S1b).

#### 2.1.3 Implementation

We implemented spline transformation using Keras (https://github.com/fchollet/keras, Chollet *et al.*, 2015), inspired by the MGCV R package (Wood, 2006). The implementation consists of three essential components: (i) a pre-processing function encodeSplines, which takes as input an array of values *x*, uniformly places B-spline bases across the range of *x* and computes $\left[b_{1}(x),\ldots ,b_{B}(x)\right]$ for each array element; (ii) a Keras layer SplineT effectively performing a weighted sum of the basis functions and (iii) a Keras regularizer SplineSmoother penalizing the squared *m*th-order differences of weights along the last dimension [Equation (3), by default second-order]. All three components are compatible with three or more dimensional input arrays *x*. Altogether this allows flexible usage of spline transformations in Keras models. The code is open source and is part of the CONCISE python package: github.com/gagneurlab/concise.

#### 2.1.4 Alternative to spline transformation: piecewise linear transformation

As an alternative to spline transformation, we consider a piecewise linear transformation achieved by stacking two fully connected layers with ReLU activation (Nair and Hinton, 2010) in-between. Formally:

(4) $$f_{PL}(x)=\sum_{k=1}^{B}w_{k}^{\left(2\right)}\text{max}(0,w_{k}^{\left(1\right)}x+b_{k}^{\left(1\right)})\;.$$

In contrast to spline transformation, the piecewise linear transformation is based on trainable basis functions ($\text{max}(0,w_{k}^{\left(1\right)}x+b_{k}^{\left(1\right)})$) and has hence more parameters. This can be of great advantage when the modeled function is compositional (Montúfar *et al.*, 2014), but can also represent a disadvantage when the modeled function is smooth.

### 2.2 Hyper-parameter tuning with Bayesian optimization

In most of the trained DNNs, we employed Bayesian optimization for hyper-parameter tuning using the Tree-structured Parzen Estimator algorithm implemented in the hyperopt python package (Bergstra *et al.*, 2013). For each trial, a hyper-parameter configuration is proposed by the Bayesian optimizer. The corresponding model is trained on the training set and evaluated on the validation set. The evaluation metric gets reported back to the optimizer. Model yielding the best performance on the validation set across all trials is selected and evaluated on the test set. This allows for a fair comparison between methods, as all the methods get equal amount of hyper-parameter tuning trials.

### 2.3 eCLIP peak prediction

#### 2.3.1 Data

RBP occupancy peaks measured by eCLIP-seq (Van Nostrand *et al.*, 2016) for human cell lines K562 and HepG2 were obtained from ENCODE version 3 (ENCODE Project Consortium, 2004). There were in total 316 experiments measuring 112 proteins. Genome assembly version GRCh38 and the corresponding GENCODE genome annotation release 25 (Harrow *et al.*, 2012) were used.

**Fig. 2.**
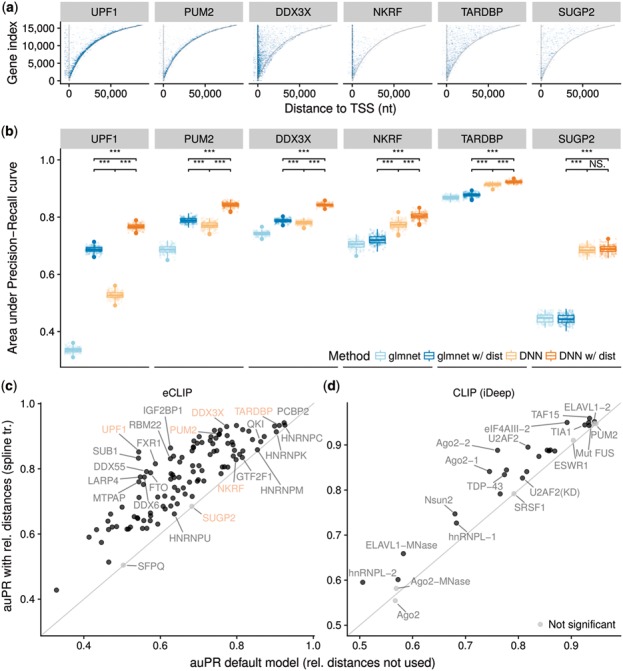
Relative distance to genomic landmarks boosts the *in vivo* prediction of RBP binding sites. (**a**) eCLIP peak distribution across all genes. Genes (*y*-axis) are sorted by their length and aligned at their start site. Color intensity represents the number of peaks per bucket (100 genes × 1000 nt) and saturates at 10 peaks per bucket. Grey lines represent gene TSS and poly(A) site. (**b**) auPR for predicting *in vivo* RBP binding sites measured by eCLIP for a subset of RBPs (6/112). Methods labelled by ‘w/dist’ rely, in addition to RNA sequence, on two positional features: distance to TSS and poly(A) site. Distribution of the auPR metric (boxplot instead of point-estimate) is obtained by generating 200 bootstrap samples of the test set and computing auPR for each of them. *** denotes *P* < 0.001 (Wilcoxon test). (**c** and **d**) Benefit of adding eight genomic landmark features with spline transformation to the (**c**) DNN model for all 112 RBPs measured by eCLIP in ENCODE and (**d**) iDeep model (Pan and Shen, 2017) for 19 RBPs across 31 CLIP experiments. Black represents statistically significant difference (*P* < 0.0001, Wilcoxon test on 200 bootstrap samples, Bonferroni correction for multiple testing)

For each RBP and each cell line (K562 and HepG2), a single set of peaks was created by intersecting the peaks from two replicate experiments, using the intersection centers as the peak midpoints (36.9% of the minimal peak number per cell line per RBP on average). This intersection was done to reduce the number of false-positive peaks, as the intersecting peaks were 2.2 times more likely to be reproduced across cell lines compared to all peaks. The positive set of peaks for each RBP was created by combining the intersected peak centers from multiple cell lines. Next, peak midpoints were overlapped with protein-coding genes. Peaks that did not map onto any annotated gene (9.9%) were discarded. Each gene–peak pair was considered as a single positive class instance. Around 94.0% of peaks mapped to a single gene and the average number of mapped genes per peak was 1.064. Example peak coverages are shown in Figure 2a. The negative set was generated by uniformly sampling within each gene four times as many locations as true binding sites in that gene. All peaks (both negative and positive) were resized to the width of 101 nt anchored at the peak center. Negative peaks that overlapped positive peaks of the same RBP were discarded.

Finally, the sequence underneath the peak was extracted, reverse-complemented for peaks from the negative strand and one-hot encoded. Relative distances from the peak center to the following eight nearest genomic landmarks on the same strand were extracted: gene TSS, transcript TSS, start codon, exon–intron boundary, intron–exon boundary, stop codon, transcript poly(A) site and gene poly(A) site. These features were further transformed with $f_{pos}(x)=\text{sign}(x)\;{\text{log}}_{10}(1+|x|)$ and min–max scaled to fit the [0,1] range. Data points from chromosomes 1, 3 were used for model validation (16%) and hyper-parameter tuning, data points from chromosomes 2, 4, 6, 8, 10 (23%) for final performance assessment and the rest for model training.

#### 2.3.2 Models

As a baseline model we considered an elastic-net model with α=0.5 (glmnet package, Friedman *et al.*, 2010) based on *k*-mer counts (k∈6,7) and positional features transformed by 10 B-spline basis functions. Smoothness regularization of B-spline features was not used. Optimal number *k* and the regularization strength were determined by 10-fold cross-validation. Models with and without the positional features were compared.

Next, we used a DNN based on two different data modalities: (i) 101 nt one-hot encoded RNA sequence beneath the peak and (ii) signed log-transformed relative distances to genomic landmarks (Supplementary Fig. S1a). DNN sequence module consisted of two 1D convolutional layers (16 filters each, kernel sizes 11 and 1, ReLU activation after each), followed by max-pooling (pooling size of 4). The positional features were either not used (DNN) or were modeled using spline transformation (DNN w/dist) in two different ways: (i) the main investigated model used a single scalar per input sequence (Supplementary Fig. S1a) and (ii) the alternative model used a vector of distances alongside the sequence (Supplementary Fig. S1b). For the main model, activation arrays of the convolutional layers (RNA sequence) and spline transformation (positional features) were concatenated and followed by two fully connected layers: a hidden fully connected layer (100 units and ReLU activation) and a final fully connected layer with sigmoid activation. For the alternative model, the activation array of spline transformation was merged with the output of the first sequence-based convolutional layer.

Batch normalization (Ioffe and Szegedy, 2015) was used after every layer and dropout (Srivastava *et al.*, 2014) before every fully connected layer. The models were optimized using ADAM (Kingma and Ba, 2014). Bayesian optimization (Section 2.2) was used to determine the optimal set of hyper-parameters for each RBP individually from 20 parameter trials, yielding the best area under the precision–recall curve (auPR) on the validation set.

### 2.4 iDeep CLIP benchmark

#### 2.4.1 Data

To compare our approach with the RBP binding site prediction model iDeep (Pan and Shen, 2017), we used the same CLIP dataset, pre-processing code and model code as Pan and Shen (2017), both provided by the authors at https://github.com/xypan1232/iDeep. The CLIP dataset contains 31 CLIP experiments measuring 19 different RBPs and was originally generated by Stražar *et al.* (2016) (available at https://github.com/mstrazar/iONMF/tree/master/datasets). Unlike eCLIP (Section 2.3.1), the peaks for each RBP from different experiments were not merged. Correspondingly, the results are always reported for each experiment individually rather than each RBP. We extended the existing set of features with relative distances to eight nearest genomic landmarks [gene TSS, transcript TSS, start codon, exon–intron boundary, intron–exon boundary, stop codon, transcript poly(A) site and gene poly(A) site], following the same procedure as for the eCLIP data (Section 2.3.1). In contrast to the eCLIP data processing, we used hg19-based GENCODE annotation v24.

#### 2.4.2 Models

As the baseline model we used the provided iDeep model with one minor modification: we replaced the softmax activation of the last layer with a sigmoid activation function (softmax is unnecessary for a binary classification task). The iDeep model is based on five different data modalities: (i) Region type, (ii) Clip-cobinding, (iii) Structure, (iv) Motif and (v) Sequence. The additional data modality introduced here—relative distance to eight genomic landmarks (Section 2.4.1)—was modeled with spline transformation using *B* = 32 basis functions and 6 output units for each feature, followed by a fully connected layer with 64 output units. This module was integrated into the iDeep model by concatenating the activations to the last hidden layer (Supplementary Fig. S1c). Spline transformation was used without smoothness regularization, because we restricted ourselves the same set of hyper-parameters as the iDeep model and have not done any hyper-parameter tuning. All models were optimized using RMSprop (Tieleman and Hinton, 2012), same as the original iDeep.

### 2.5 Branchpoint prediction

#### 2.5.1 Data

**Fig. 3.**
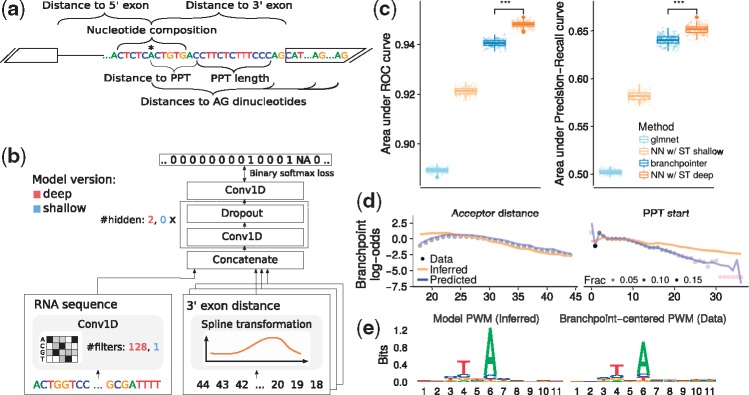
Spline transformation improves branchpoint prediction: (**a**) Features for branchpoint prediction designed by Signal *et al.* (2016) (adapted from Signal *et al.*, 2016). (**b**) NN model architectures (deep and shallow) for branchpoint prediction developed here. For each predicted binary class (1 = high-confidence branchpoint, NA = ignored low-confidence branchpoint, 0 = else), the model takes as input 11 nt sequence window and 9 position-related features. (**c**) auROC and auPR bootstrap distribution (*n* = 200) for branchpoint prediction on the test set. Our NN models, spline transformation shallow (NN w/ST shallow) and spline transformation deep (NN w/ST deep), are compared to the state-of-the-art model branchpointer (Signal *et al.*, 2016) and an elastic-net baseline using the same features. *** denotes *P* < 0.001 (Wilcoxon test). (**d**) Fraction of branchpoints per position for the two most important features in the log-odds scale (black dot, outlier shown in red) compared to the shallow NN model fit: inferred spline transformation (orange) and predicted branchpoint log-odds (blue). (**e**) Information content of the shallow NN convolutional filter transformed to the PWM and the branchpoint-centered PWM (Section 2)

Branchpoint prediction is a binary classification task to predict measured high-confidence branchpoints in introns, 18–44 nt upstream of the 5ʹ intron–exon boundary. The same dataset and pre-processing procedure was used as described in the work of Signal *et al.* (2016). Briefly, high-confidence annotated branchpoints from Mercer *et al.* (2015) were used to generate the positive set. Negative set comprises of positions not annotated as high- or low-confidence branchpoints in the work of Mercer *et al.* (2015). This yields in total 52 800 positive and 933 739 negative examples. Signal *et al.* (2016) designed and used the following features in the classification model (Fig. 3a): 11 nt sequence window around the position encoded as dummy variables, distances to the first five canonical AG dinucleotides downstream, distance to the poly-pyrimidine tract (PPT) and its length, distance to the associated 3ʹ exon and distance to the nearest 5ʹ exon located on the same strand. GENCODE v12 (Harrow *et al.*, 2012) was used for genome annotation. Using the code provided by Signal *et al.* (2016) (https://github.com/betsig/splice_branchpoints), we were able to reproduce the results of Signal *et al.* (2016). The only major change to the pipeline was to a priori set aside points from chromosomes 4, 5, 6, 7, 8 and X (21% of all the data) as a test set. The test set was only used to test the predictive performance of our models and not to tune the hyper-parameters as done in Signal *et al.* (2016). Exact code changes can be tracked in our forked repository (https://i12g-gagneurweb.informatik.tu-muenchen.de/gitlab/avsec/splice_branchpoints).

#### 2.5.2 Models

All the models use the same set of features as Signal *et al.* (2016).

**branchpointer**: Branchpoint prediction model developed by Signal *et al.* (2016). It is a combination of two stacked models: support vector machine (SVM) with ‘rbfdot’ kernel and a gradient-boosted decision trees model, both from the caret R package (Kuhn, 2008).

**glmnet**: Logistic regression with elastic-net regularization using the glmnet R package (Friedman *et al.*, 2010) with parameters *α* = 0.5 and regularization strength determined by 5-fold cross-validation on the training dataset.

**NN**: DNN developed here (Fig. 3b). For computational efficiency, the model predicts the branchpoint class for all 27 positions in an intron simultaneously, while using the same parameters for each position. Specifically, the models take as input one-hot encoded 37 nt long RNA sequence and 9 position-related features, each as an integer array of length 27. Parameter sharing across 27 positions within an intron is achieved with 1D convolutions using kernel size of 1. The only exception is the first convolutional layer processing RNA sequence where kernel size of 11 is used. That way, the set of features for predicting the branchpoint class at a single position is exactly the same as for branchpointer and positions are completely independent of each other.

The nine positional features were transformed either with: (i) spline transformation or (ii) piecewise linear transformation. Moreover, two levels of model complexity were compared: ‘shallow’ and ‘deep’. They differ in the number of convolutional filters, number of hidden layers (Fig. 3b) and also in the weight initialization for the first sequence-based convolutional layer: ‘shallow’ models were initialized with the position-specific scoring matrix of the high-confidence branchpoints derived from the training set and ‘deep’ models were initialized with the (random) glorot-uniform initialization. In total, four different model architectures were used. Hyper-parameters were tuned for each of the four NN classes individually using Bayesian optimization (Section 2.2).

#### 2.5.3 Position weight matrix analysis

Weights of the convolutional filter *w_ij_* were converted to a position weight matrix (PWM) by

$${\text{PWM}}_{ij}=\frac{b_{i}\;\text{exp}\;(w_{ij})}{\sum_{i}b_{i}\;\text{exp}\;(w_{ij})}\;,$$

where i∈{A,C,G,T} is the nucleotide identity and *b_i_* is the background probability (*A*: 0.21, *C*: 0.25, *G*: 0.20 and *T*: 0.34 in the branchpoint dataset). Note that we are denoting T also as Uracil. Branchpoint-centered PWM was created from 11 nt long sequences centered at the high-confidence branchpoints from Mercer *et al.* (2015).

### 2.6 Table 1 description

Table 1 shows the average auPR for the following models:

**Table 1.** Test accuracy (auPR) of investigated models across all tasks (Section 2)

Task	State-of-the-art model	glmnet	glmnet + dist	DNN	DNN +dist(PL)	DNN +dist(ST)
eCLIP	/	0.557	0.677	0.663	0.743	0.776***
CLIP	iDeep: 0.800	0.555	0.660	0.625	0.804	0.835***
branchpoint	branchpointer: 0.640	0.419	0.502	0.478	0.645	0.651***

*Note*: Significance of the improvement for DNN + dist(ST) against DNN + dist(PL) was assessed across all RBPs for eCLIP and CLIP tasks and across the 200 bootstrap samples for the branchpoint task.

*** *P* < 0.001, Wilcoxon test, paired for eCLIP and CLIP.

**Fig. 4.**
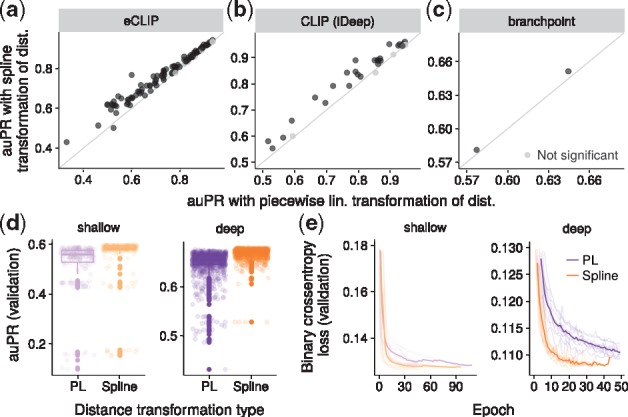
Spline transformation outperforms piecewise linear transformation in terms of generalization accuracy, hyper-parameter robustness and training efficiency. (**a–c**) Test accuracy (auPR) comparing spline transformation to piecewise linear transformation for all the tasks presented in the paper (Figs 2c and d and 3c). Black represents statistically significant difference (*P* < 0.0001, Wilcoxon test on 200 bootstrap samples, Bonferroni correction for multiple testing). (**d** and **e**) Training and hyper-parameter tuning metrics for the branchpoint task. PL, piecewise linear. (**d**) Validation accuracy (auPR) of all the hyper-parameter trials. (**e**) Training curves (validation loss per epoch) of 10 best hyper-parameter trials (transparent lines) and their average (solid line)

DNN + dist(PL) and DNN + dist(ST): DNNs using sequence and distance features. Distance features were processed either using piecewise linear transformation or spline transformation. For eCLIP and CLIP, the average axis values from Figure 4c were used. For branchpoint, the auPR of the deep model from Figure 4c was used.

DNN: DNNs using only sequence as input. For eCLIP, the average *x*-axis value from Figure 4c was used. For CLIP, the iDeep model was retrained for all 31 RBPs using only the sequence data modality. For branchpoint, the DNN model using only the sequence as input was retrained and its hyper-parameters optimized as for the original model.

glmnet and glmnet + dist: Elastic-net logistic regression analogs of the DNN and DNN + dist(ST) models. For eCLIP and CLIP, the models follow the same approach as those shown in Figure 2b, but use 6-mers for the sequence features and all eight distance features in the glmnet + dist model. For branchpoint, the glmnet value in Figure 3c was used for the glmnet + dist and the same model was retrained using only sequence features for the glmnet column.

## 3 Results

### 3.1 Relative distance to genomic landmarks improves *in vivo* RBP binding prediction

We first investigated the benefit of modeling effects of position with respect to genomic landmarks for the task of predicting *in vivo* binding sites of RBPs. We used a large and consistently generated dataset of eCLIP data for 112 RBPs from the ENCODE project (Section 2, Van Nostrand *et al.*, 2016).

For a representative detailed investigation, we first focused on six RBPs with more than 10 000 peaks and exhibiting various peak distributions along genes (Fig. 2a). Comparing the relative positions within genes between the binding and non-binding sites (Supplementary Fig. S2), we selected two RBPs with high enrichment toward the TSS (DDX3X, NKRF, *t*-test comparing positions of binding sites versus non-binding sites $P<10^{-100}$), two RBPs showing high enrichment toward the poly(A) site (UPF1 and PUM2, $P<10^{-100}$) and two RBPs showing no significant positional preference (TARDBP, SUGP2, *P* > 0.5). We next asked what the contribution of using a DNN on the one hand and of modeling positional effects on the other hand for the task or predicting eCLIP peaks was. To this end, we fitted four models (Section 2): (i) an elastic-net logistic regression based on *k*-mer counts from 101 nt sequence around the candidate peak as a non-deep supervised learning algorithm (glmnet), (ii) an extension of the latter model that also included relative distance to gene TSS and poly(A) site transformed by spline transformation (glmnet w/dist), (iii) a DNN based on the 101 nt sequence around the candidate peak (DNN) and (iv) an extension of the latter model with spline transformation of relative distance to gene TSS and poly(A) site (DNN w/dist, Supplementary Fig. S1a).

For each of the six RBPs, the DNNs yielded a significantly larger auPR (a metric between 0 and 1, the larger, the better) compared to their corresponding elastic-net based models (Fig. 2b). Moreover, modeling positional effects significantly improved the performance for all four RBPs showing positional preference. In three out of four cases (UPF1, PUM2 and DDX3X), the glmnet model even outperformed the DNN model lacking positional features. Overall, DNN with distance was always the most performant model. Although its training took typically 10–100 times longer than elastic-net (Supplementary Fig. S3), it remained practical (<1000 s on a desktop CPU using four threads) and did not take longer than DNN without distance. These results show the importance of modeling positional effects for predicting RBP binding peaks and the power of combining this approach with DNNs.

Next, we extended our set of positional features in DNN w/dist to eight genomic landmarks (nearest gene TSS, transcript TSS, start codon, exon–intron boundary, intron–exon boundary, stop codon, transcript poly(A) site, gene poly(A) site; Supplementary Fig. S4) and compared it with DNN across all the 112 RBPs. Using relative distances increased the auPR by up to 0.31 (UPF1, from 0.54 to 0.85), on average by 0.11 (from 0.66 to 0.77, $P<10^{-16}$ paired Wilcoxon test, Fig. 2c). Altogether, 110 RBPs showed significant auPR increase and none a significant decrease (P<0.0001, Wilcoxon test, Bonferroni correction for multiple testing). Similar result was also obtained with an alternative version of the DNN w/dist model using a vector of distances along the sequence rather than a single value per sequence. Since the performance of the two DNN w/dist variants differed only by 0.0014 on average (Supplementary Fig. S5), we used the simpler model (scalar per sequence) for the downstream analysis.

As expected, RBPs with the smallest auPR increase did not exhibit positional preference for any of the eight genomic landmarks, in contrast to the RBPs with the largest auPR increase (Supplementary Fig. S4). Among the 10 RBPs with the smallest auPR increase were 4 members (HNRNPM, HNRNPC, HNRNPK and HNRNPU) of the Heterogeneous nuclear ribonucleoprotein particle, which is a general nuclear complex binding precursor RNAs (Choi *et al.*, 1986).

The models were robust to exclusion of individual distance features (Supplementary Fig. S6), even for the RBPs with the most striking positional preference (Supplementary Fig. S4). This likely reflects redundancy among the distances. For instance, positional preference towards the gene end can well be captured by distance towards the closest poly(A) site or even the closest stop codon. This property makes these models robust to the exact choice of genomic landmarks. However, we note that it also renders model interpretation difficult.

To further validate our observations from the eCLIP data, we extended the current state-of-the-art model for RBP binding site prediction—iDeep (Pan and Shen, 2017) with the same eight genomic landmark features. iDeep is a DNN trained and evaluated on a CLIP dataset of 19 proteins measured by 31 experiments created by Stražar *et al.* (2016). It does not model distances to genomic landmarks quantitatively. However, it is based on indicator features for five gene regions (exon, intron, 5ʹ UTR, 3ʹ UTR, CDS) for each nucleotide in the classified sequence. Since iDeep was already implemented in Keras, extending it with our spline transformation module could be done easily (Section 2). When we added the eight positional features with spline transformation on top of iDeep, the auPR increased by 0.036 ($P=3.1\times 10^{-8}$, paired Wilcoxon test) and area under receiver operating characteristic curve (auROC) by 0.017 ($P=9.3\times 10^{-10}$). The auPR improved significantly for 24 out of 31 experiments and has not significantly decreased for any experiment (Fig. 2d). This shows that the quantitative distances, and not just binary indicators, are useful predictive features for RNA binding sites. Moreover, this application demonstrates how spline transformation modules can enhance existing deep learning models.

Altogether, these results demonstrate that relative distance to genomic landmarks is an important feature for predicting *in vivo* RBP binding events and show that our spline transformation module provides a practical way to include this information in DNNs.

### 3.2 Spline transformation in a DNN improves state-of-the-art branchpoint prediction

We then asked whether spline transformation in a DNN could improve prediction accuracy for tasks where the effect of the distance to genomic landmarks has already been exploited by non-deep learning methods. To this end, we considered the prediction of splice branchpoint. The first reaction of splicing is the attack of a 2ʹ hydroxyl group of an intron adenosine on the 5ʹ splice site phosphodiester bond (Ruskin *et al.*, 1985). This intron adenosine is located typically between 18 and 44 nt 5ʹ to the acceptor site (Mercer *et al.*, 2015). It is named branchpoint, because it is bound on its 2ʹ hydroxyl group, leading to a lariat form of the spliced-out intron. Mapping branchpoints experimentally has been difficult because of the very short half-life of lariats. Computational predictions of branchpoints have been also difficult because their sequence context is degenerate (Gao *et al.*, 2008).

Current state-of-the art model to predict human branchpoints is *branchpointer* (Signal *et al.*, 2016), an ensemble model of SVM and gradient boosting machine trained on a set of 42 095 mapped high-confidence branchpoints from Mercer *et al.* (2015). In addition to the sequence context, *branchpointer* uses 11 different positional features: distances to the first five downstream AG dinucleotides, distance to the PPT and its length, distance to the associated 3ʹ and 5ʹ exon (Fig. 3a, Section 2).

Using the provided code and some minor modifications (Section 2), we were able to reproduce the results of branchpointer and obtained very similar performance metrics as originally reported: auROC of 0.940 (paper: 0.941) and auPR of 0.640 (paper: 0.617) (Fig. 3c). Training a DNN with spline transformation module for positional features (Fig. 3b) significantly outperformed *branchpointer* with auROC of 0.949 and auPR of 0.651 ($P<2.2\times 10^{-16}$, Wilcoxon test, Fig. 3c). This result is consistent with general improved performance of DNNs over alternative supervised learning models and shows the strength of spline transformation. It also yields to the most accurate predictor of human branchpoints to date.

### 3.3 Shallow architecture yields an interpretable branchpoint model while still delivering good predictive performance

When model interpretation rather than mere prediction is desired, shallow neural networks are preferred over DNNs because their coefficients can be directly interpreted. To investigate such a use case, we trained a shallow version of our neural network (NN w/ spline transformation shallow, Section 2) for branchpoint prediction. As expected, the shallow model is not able to compete with its deeper version or *branchpointer*. Nevertheless, it performs well compared to an elastic-net logistic regression (Fig. 3c).

Predicted positional effects in the shallow model (‘Predicted’ in Fig. 3d, Supplementary Fig. S7) closely resembled the distributions of branchpoint distances to all genomic landmarks (‘Data’ in Fig. 3d, Supplementary Fig. S7). In addition to the distances, the single convolutional filter in our shallow model captured the expected sequence preference of branchpoints (Fig. 3e, Mercer *et al.*, 2015).

Altogether, these analyses of branchpoint prediction demonstrate the versatility of the spline transformation module. The spline transformation module can be used to increase predictive power in conjunction with DNN. It can also be employed in shallow and interpretable models.

### 3.4 Spline transformation is more robust to hyper-parameter choices, trains faster and yields better predictive performance than piecewise linear transformation

The most widely used transformations in deep leaning currently are compositions of linear transformation and ReLUs, defined as ReLU(x)=max(0,x). Composition of those lead to piecewise linear transformations (Section 2). Although PL functions can approximate any function, this can be at the cost of introducing much more parameters. Also PL functions are not smooth.

To inspect the benefit of spline transformation compared to the default modeling choice in deep learning, we replaced the spline transformation module with piecewise linear transformation (Section 2) in all three studied tasks. As for the spline transformation, we used the same number of single output units, equivalent number of hidden units and the same hyper-parameter optimization strategy for each task. The observed predictive performance of spline transformation was consistently better across all three tasks (Fig. 4a–c): (i) for eCLIP the auPR improved on average by 0.033 ($P<2.2\times 10^{-16}$, paired Wilcoxon test) and auROC by 0.018 ($P<2.2\times 10^{-16}$); (ii) for the iDeep CLIP benchmark dataset, the auPR improved on average by 0.032 ($P=1.8\times 10^{-8}$) and auROC by 0.017 ($P=2.8\times 10^{-9}$); (iii) for branchpoint (deep model), the auPR improved by 0.006 ($P<2.2\times 10^{-16}$, Wilcoxon test on 200 prediction bootstrap samples) and auROC by 0.001 ($P<2.2\times 10^{-16}$).

Focusing on the branchpoint prediction task, we compared the validation accuracies of all hyper-parameter trials between spline transformation and piecewise linear transformation (Fig. 4d). Spline transformation had fewer trials with poor performance and globally smaller performance variation. This suggests that spline transformation is more robust to parameter initialization and hyper-parameter choices like the learning-rate. Additionally, we inspected the training curves of the top 10 hyper-parameter trials (Fig. 4e). While the DNN with spline transformation on average trained in 20 epochs, piecewise linear transformation required more than 50 epochs. Moreover, spline transformation, which has fewer parameters, required 4% less training time per epoch for the deep and 36% for the shallow model on average (Supplementary Fig. S8). Altogether these results show that spline transformations generalize better, are more robust, are train in fewer steps than piecewise linear transformations for the class of problems we investigated.

### 3.5 Contribution of model components to the investigated tasks

To delineate the contribution of DNNs, of distance features and of the spline transformation, we summarized the test accuracy of models differing by only one of these components across all the investigated tasks in Table 1. Overall, distance information always substantially improved the performance of both, elastic-net logistic regression models and DNNs. Moreover, DNNs systematically outperformed the elastic-net logistic regression models. For each task, the best model was a DNN with distance information integrated by spline transformation [DNN + dist(ST)].

## 4 Discussion

Here we have introduced spline transformations, a module for neural networks, and demonstrated that it is an effective way to model relative distance to genomic landmarks. Spline transformations allowed us to improve the state-of-the-art prediction accuracy of splice branchpoint and *in vivo* RBP binding affinity. On the latter task, the use of relative distance to genomic landmarks in a neural network is novel. Moreover, we have shown that spline transformation in a shallow network can uncover the positional effects of *cis*-regulatory elements.

We provide spline transformation as an open-source Keras components. We have shown how to combine it with existing models and improve their performance. Compared to a two-layer neural network with ReLU activations—piecewise linear transformation, spline transformation offers better prediction accuracy, is more robust to initialization and trains faster. This is not surprising as the relative positional features tend to affect the response variable in a smooth fashion, which is exactly the class of functions spline transformation is able to represent with very few parameters.

In addition to external positions studied here, spline transformation can also be used to model *internal* positions, which are positions within the sequence. In that case, the array index *i* along the spatial dimension of the 1D convolutional layer activation *a_ij_* serves as the relative distance feature. That way, weights in the recently introduced weighted sum pooling layer (Shrikumar *et al.*, 2017) can be parametrized by spline transformation: $w_{ij}=f_{S}(i)$. Note that this applies also to the separable fully connected layer (Alexandari *et al.*, 2017), which can be reformulated as 1D convolution with kernel size of 1 followed by a weighted sum pooling layer. Altogether, using spline transformation for modeling internal position reduces the number of parameters in the network even further.

One limitation of spline transformation is that scale of the input features (e.g. log or linear) remains important and has to be chosen upfront, because spline knots are placed uniformly across the whole range of feature values. We suggest users to perform pre-processing investigations to identify the most appropriate scales for the problem at hand. Moreover, the current implementation of spline transformation is not able to model the interaction between variables directly. While this interaction is still captured by the downstream fully connected layers, a more appropriate solution might be to use multi-dimensional B-splines. A further research direction is the estimation of confidence bands for the inferred spline transformation function. Confidence bands for spline estimates are available in the context of generalized additive models (Hastie and Tibshirani, 1990). We have recently shown how this can be used to perform differential occupancy analysis of ChIP-seq data (Stricker *et al.*, 2017). Confidence bands would allow deriving statistically supported claims about the positional effects of *cis*-regulatory elements.

We have demonstrated the power of using spline transformations for modeling effects of distances to genomic landmarks. However, the spline transformation module is more general. It could be used to transform any other relevant scalar. Relevant scalars for *cis*-regulatory elements include conservation scores, modifications such as methylation rates, experimental measures such as occupancies by factors or nucleosomes. Hence, we foresee spline transformations as a useful generic tool for modeling of *cis*-regulatory elements with neural networks.

## Supplementary Material

Supplementary Figures
