# Supplementary material for: Modeling positional effects of regulatory sequences with spline transformations increases prediction accuracy of deep neural networks
Source: Bioinformatics. 2017 Nov 16;34(8):1261–9. doi: 10.1093/bioinformatics/btx727 (PMC5905632; doi:10.1093/bioinformatics/btx727)
Supplement: Supplementary Figures [file btx727_supplementary_figures.pdf]

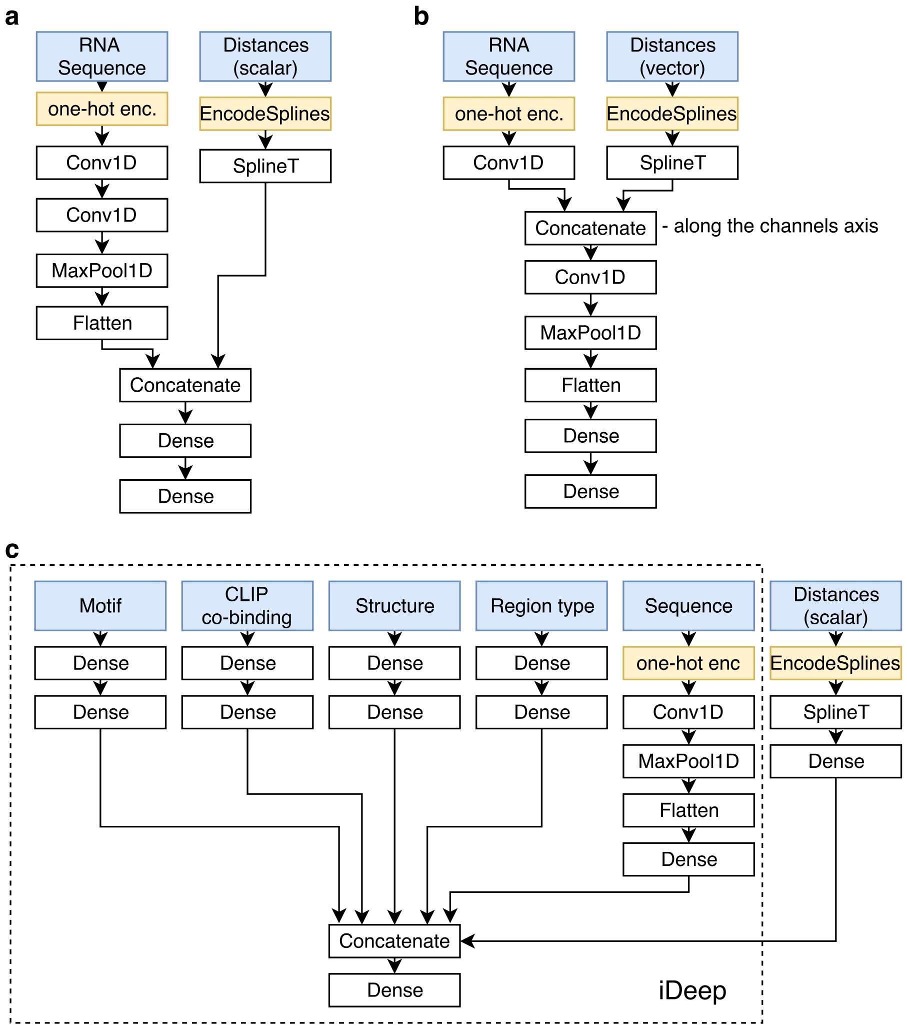

Figure S1. CLIP model architectures: **(a,b)** eCLIP models. They differ in the way distance is used. In (a), only a single value (scalar) per input sequence is used. In (b) the distance is used as a vector along the sequence (one value per nucleotide). **(c)** iDeep model extended with spline transformation. Blue rectangles denote the input data modality, yellow rectangles denote the pre-processing function and white rectangles denote the neural-network layers using Keras notation. Batch normalization and dropout layers are not shown here but are specified in the Methods section.

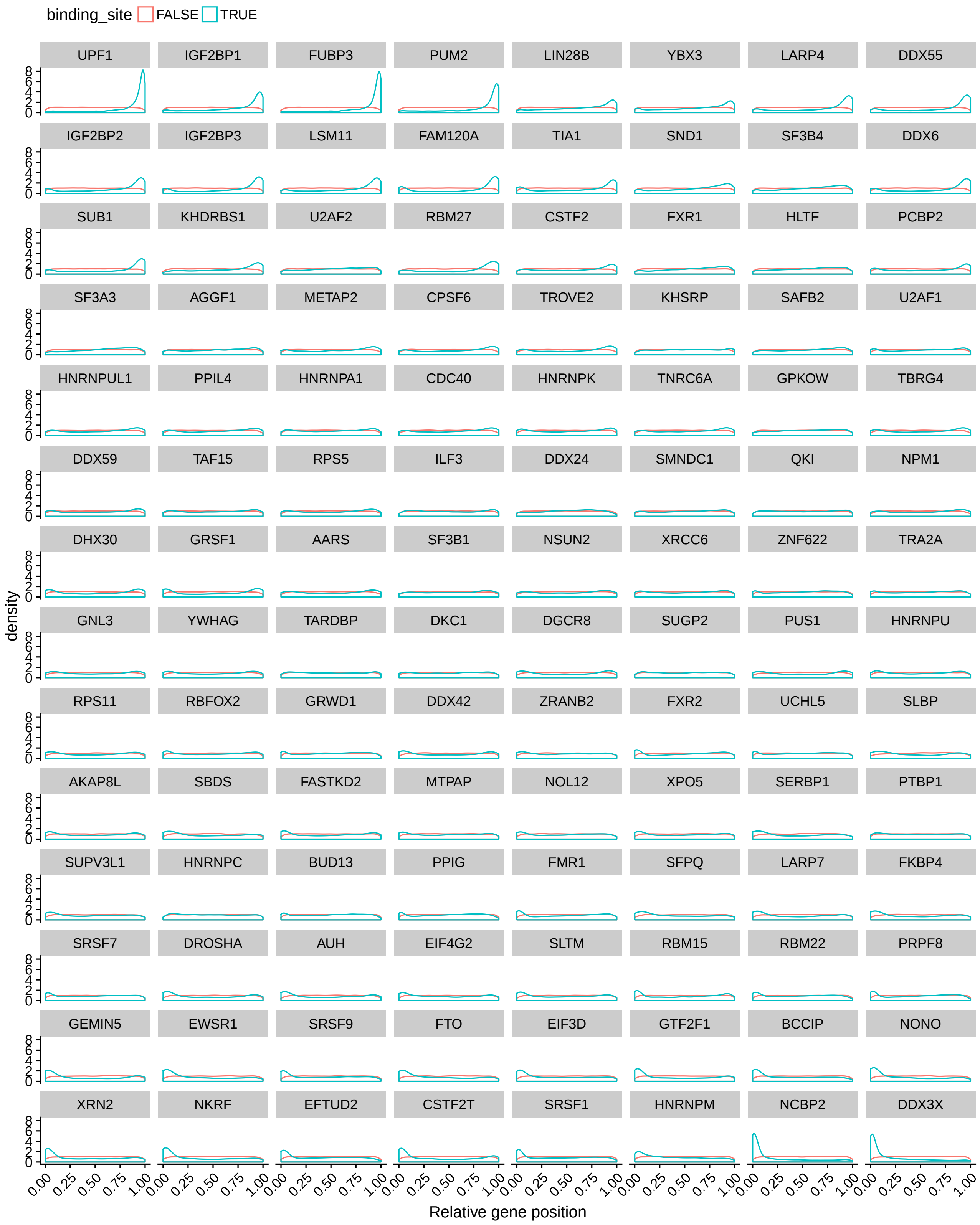

Figure S2. Relative genes positions for 112 RBPs measured by eCLIP. RBPs are sorted by the t-test statistic comparing relative genes positions of binding to non-binding sites.

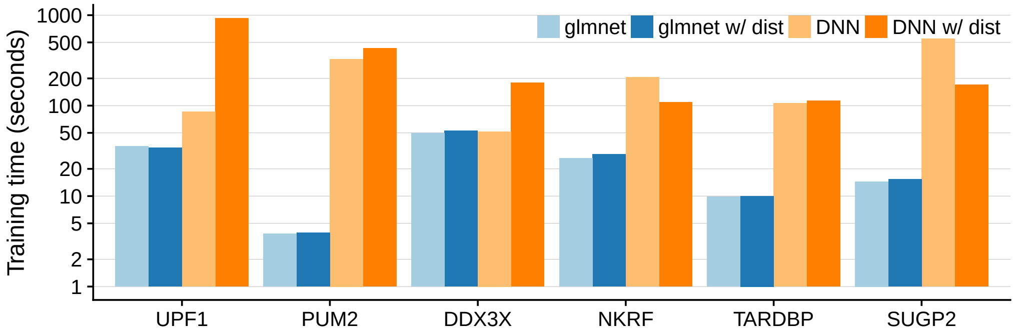

Figure S3. Training time of models shown in Figure 2b using 4 virtual threads of an Intel Core i7-6700 CPU.

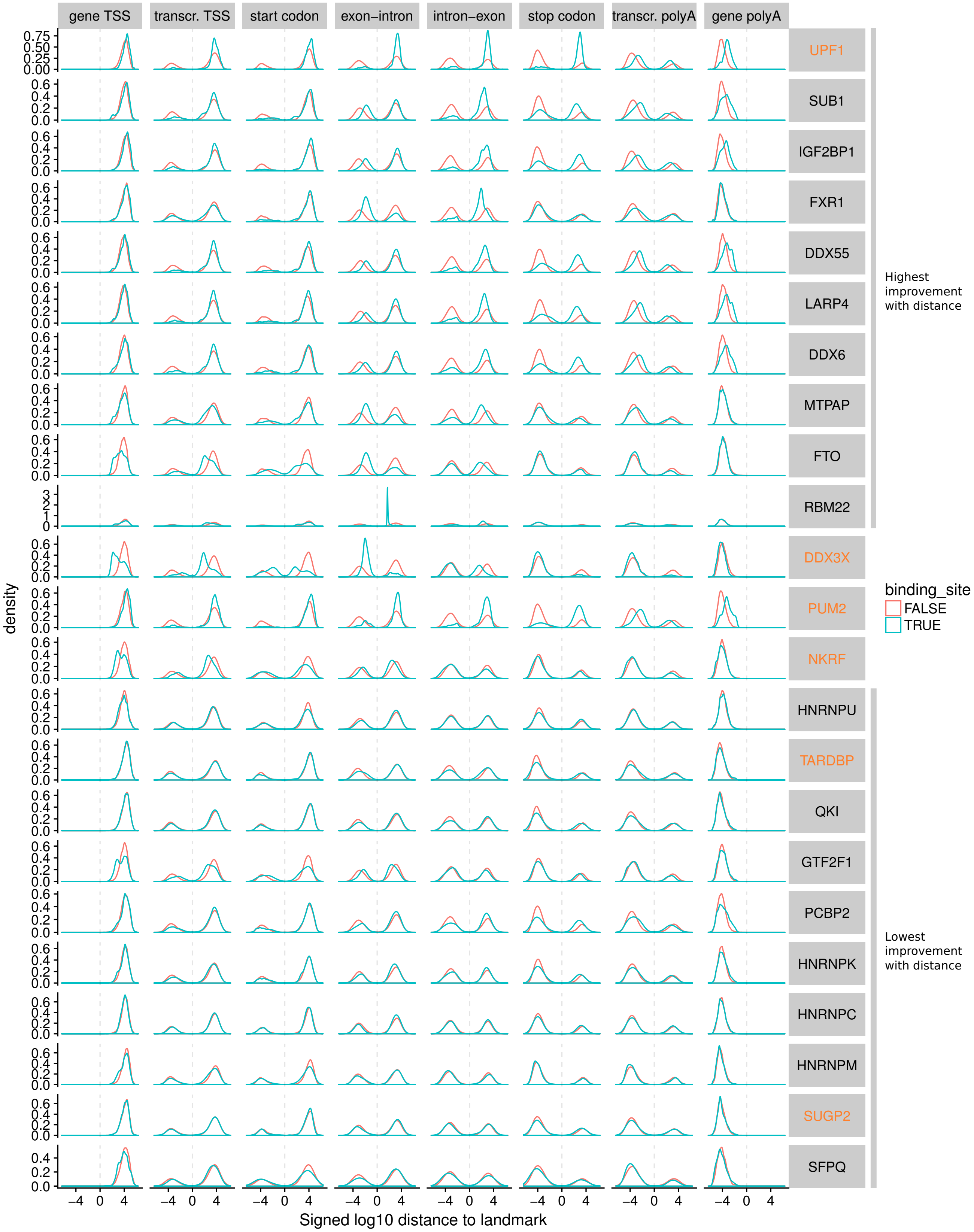

Figure S4. Relative distance to all 8 considered genomic landmarks for eCLIP peaks. Only RBPs labelled in Figure 2c are shown.

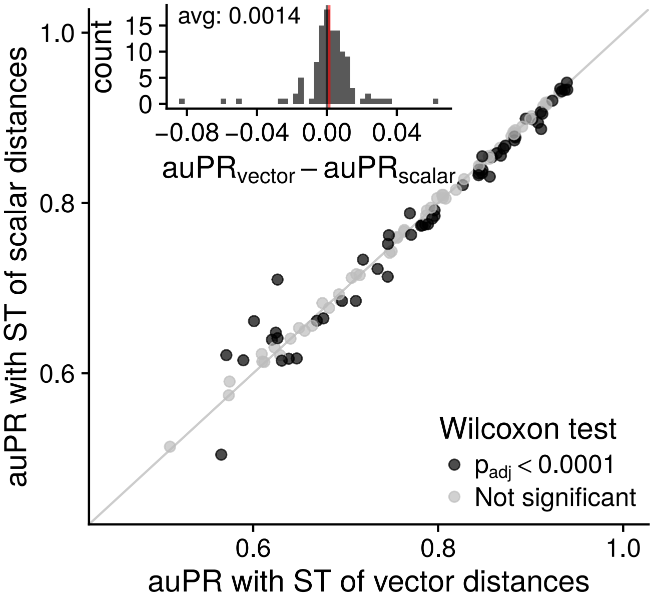

Figure S5. Test-accuracy (auPR) comparing two different variants of the eCLIP DNN w/ dist model: the main model using a single scalar per input sequence (y-axis, architecture shown in Figure S1a) and the alternative model using a vector of distances alongside the sequence (x-axis, architecture shown in Figure S1b). Black represents statistically significant difference ($P < 0.0001$, Wilcoxon test on 200 bootstrap samples, Bonferroni correction for multiple testing). Histogram within the scatterplot shows a distribution of performance differences and the average value.

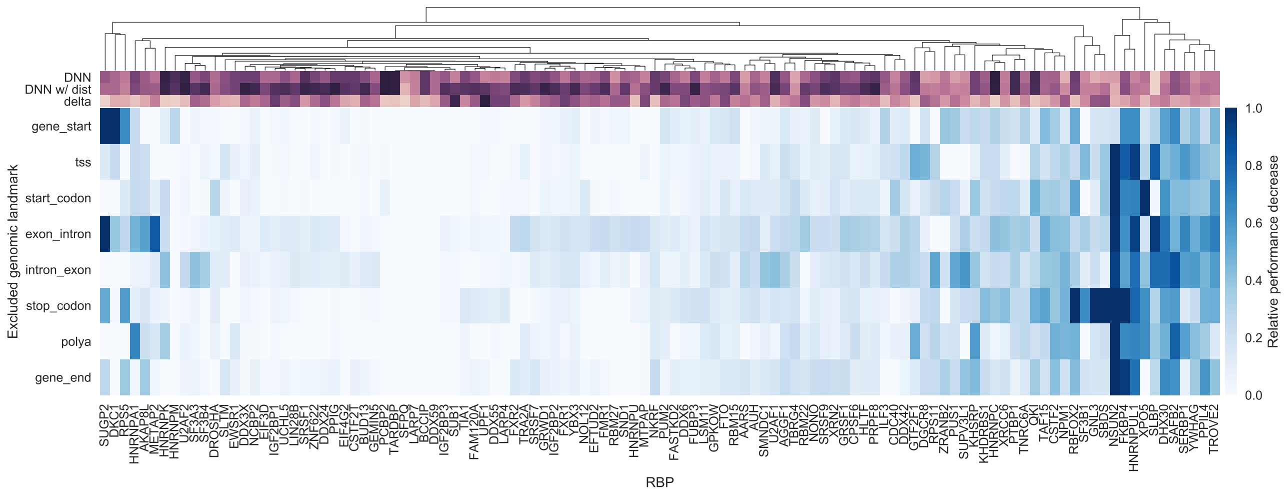

Figure S6. Relative test-accuracy (auPR) decrease when excluding individual features from the eCLIP DNN w/ dist model. A relative decrease of 1 means that the test-accuracy decreased to the accuracy of the sequence-only model (DNN) and a relative decrease of 0 means the test-accuracy remained unchanged (i.e. equals the DNN w/ dist model accuracy). Top rows in purple shows the auPR of the DNN, DNN w/ dist model and their difference. Darker red denotes a higher auPR. Relative decrease variations for the 11 rightmost RBPs should be interpreted with care since both the overall performance and the added value of including distance is low.

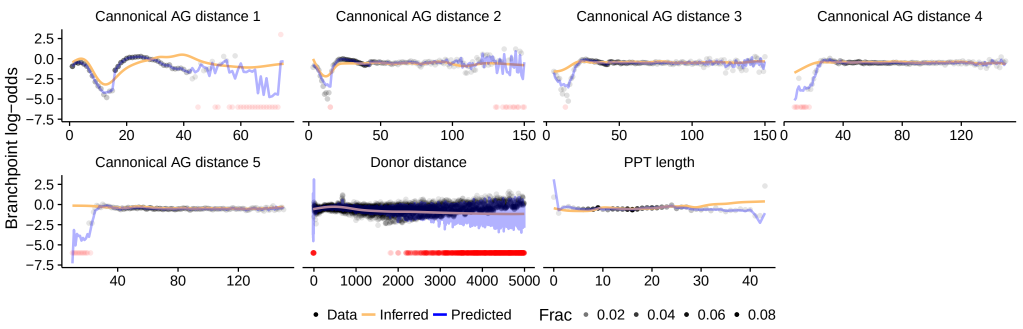

Figure S7. Fraction of branchpoints per position for the remaining seven features in log-odds scale (black dot, outlier shown in red) compared to the shallow NN model fit: inferred spline transformation (orange) and predicted fraction of branchpoint per position (blue).

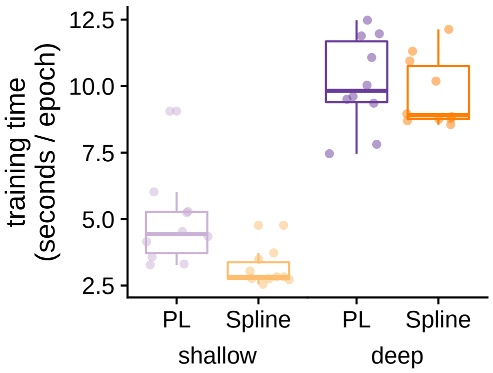

Figure S8. Training time per epoch of models shown in Figure 4e using 4 virtual threads of an Intel Core i7-6700 CPU.
